# P-1055. Anti-Toxin B Neutralizing Monoclonal Antibody AZD5148 Provides Protection in a *Clostridioides difficile* Gnotobiotic Piglet Model

**DOI:** 10.1093/ofid/ofae631.1244

**Published:** 2025-01-29

**Authors:** Christine Tkaczyk, Denise Dayao, Donald Girouard, Victoria Godfrey, Adam Gamson, Ann Marie Stanley, Oleg Stepanov, Antonio DiGiandomenico, Bret R Sellman, Saul Tzipori

**Affiliations:** AstraZeneca BioPharmaceuticals R&D, Gaithersburg, Maryland; Tufts University, North Grafton, Massachusetts; Tufts University, North Grafton, Massachusetts; AstraZeneca, Gaithersburg, Maryland; Astra Zeneca, Gaithersburg, Maryland; Translational Medicine, Vaccines & Immune Therapies, BioPharmaceuticals R&D, AstraZeneca, Gaithersburg, MD, USA, Gaithersburg, Maryland; AstraZeneca, Gaithersburg, Maryland; Astra Zeneca, Gaithersburg, Maryland; AstraZeneca, Gaithersburg, Maryland; Tufts University, North Grafton, Massachusetts

## Abstract

**Background:**

*Clostridioides difficile* infection (CDI) cause mild to severe diarrhea and can be life threatening. Because toxin B is a clinically validated target for CDI, we have generated an anti-toxin B neutralizing monoclonal antibody (mAb) AZD5148. We previously demonstrated that AZD5148 administered at 10, 1 and 0.5 mg/kg prevents severe disease development in a gnotobiotic piglet model, which recapitulates the clinical signs and gross pathology observed in human CDI, we compared the protective efficacy of AZD5148 with the clinically approved anti-toxin B mAb bezlotoxumab.

**Methods:**

Piglets were born by C-section and placed in an individual sterile isolator. Animals were first injected intraperitoneally with 0.5 mg/kg of *Clostridioides difficile* (*C. difficile)* mAbs or control IgG (c-IgG) and infected orally 24 hours later with *C.difficile* spores of NAP1/027/BI strain UK6. Weight and stool scores were recorded daily. Non-infected animals were used as comparators. Necropsy was performed after 7 days for colon gross pathology scoring and tissue histopathology analysis. Anti-toxin B pharmacokinetics were measured in sera by ELISA at days 0, 3 and 7.

**Results:**

Infection with *C. difficile* reduced weight gain compared to noninfected littermates. All infected animals developed mild to moderate diarrhea after 3 days. Piglets treated with c-IgG or bezlotoxumab exhibited signs of severe disease as measured by diarrhea color and texture. Gross pathological examination of colons at necropsy for c-IgG treated-pigs suggested that animals developed similar pathology to that observed during human CDI with mild to severe hemorrhage of the spiral colon, mesocolonic edema and pseudomembrane formation on the descending colon at day 7, along with rectal prolapse in some cases. No significant difference was observed between the c-IgG and bezlotoxumab groups. In contrast, AZD5148 reduced signs of disease severity as measured by diarrhea scores, gross pathology scoring of descending and spiral colons and damage to the gut epithelium along with inflammation.

**Conclusion:**

These data suggest that AZD5148 may offer some therapeutic benefit in preventing CDI in humans.

**Disclosures:**

**Christine Tkaczyk, PhD**, Astra Zeneca: stocks as employee **Victoria Godfrey, MS**, Astra Zeneca: stocks as employee **Adam Gamson, MS**, Astra Zeneca: stocks as employee **Ann Marie Stanley, PhD**, Astra Zeneca: stocks as employee **Oleg Stepanov, MS**, Astra Zeneca: stocks as employee **Antonio DiGiandomenico, PhD**, Astra Zeneca: stocks as employee **Bret R. Sellman, PhD**, Astra Zeneca: stocks as employee

